# Aortic Valve Annular Parameters in Idiopathic Hypereosinophilic Syndrome—A Detailed Investigation from the Three-Dimensional Speckle-Tracking Echocardiographic MAGYAR-Path Study

**DOI:** 10.3390/biomedicines14030614

**Published:** 2026-03-10

**Authors:** Attila Nemes, Nóra Ambrus, Zita Borbényi

**Affiliations:** Department of Medicine, Albert Szent-Györgyi Medical School, University of Szeged, H-6725 Szeged, Hungary; ambrusnora@gmail.com (N.A.);

**Keywords:** echocardiography, hypereosinophilic syndrome, aortic valve annulus, three-dimensional, speckle-tracking

## Abstract

**Introduction**: Hypereosinophilic syndrome (HES) is characterized by a sustained elevation in eosinophil counts in the blood and subsequent eosinophil-mediated tissue or organ damage. While the aortic valve is recognized as a pivotal determinant of central hemodynamics, aortic valve annular (AVA) involvement in a series of HES patients has not yet been investigated. Therefore, this investigation aimed to determine potential abnormalities in the AVA dimensions and dynamics in patients with HES and to assess their associations with function of the left ventricle (LV). **Methods**: The present retrospective study initially consisted of 17 HES patients; however, one subject was excluded because of the suboptimal image quality. The final group of HES patients comprised 16 cases (mean age: 59.7 ± 12.6 years; 11 males). A group of 21 gender- and age-matched healthy subjects served as the controls (mean age: 54.0 ± 4.9 years; 12 males). **Results**: Among the 16 HES patients, the end-systolic AVA area was greater than, equal to, or smaller than the end-diastolic AVA area in four (25%), three (19%) and nine (56%) cases, respectively. In the matched control group, these proportions proved to be 12 (57%), one (5%) and eight (38%), respectively. No significant differences were found in the end-systolic and end-diastolic AVA minimum and maximum perimeters, areas or diameters between the HES patients and the matched controls, suggesting that no absolute AVA dilation was observed, despite an altered end-diastolic—end-systolic AVA area distribution. The AVA plane systolic excursion (AAPSE) was significantly decreased in the HES patients (0.91 ± 0.29 cm vs. 1.12 ± 0.24 cm, *p* = 0.05). Among the basal LV strain parameters, only the LV longitudinal strain (LS) was significantly impaired in the HES patients (−16.63 ± 4.99% vs. −21.62 ± 4.76%, *p* < 0.05). **Conclusions**: In HES patients, the AVA is not significantly dilated. However, a greater end-diastolic AVA area is found to be more frequently present compared with age- and gender-matched healthy controls. In addition, AAPSE and basal LV-LS are significantly reduced in HES patients.

## 1. Introduction

A diagnosis of idiopathic HES is confirmed when, in addition to meeting the thresholds for peripheral blood hypereosinophilia, there is clear evidence of organ injury resulting from tissue-based eosinophilia, and all alternative primary or secondary causes for these findings have been ruled out [1,2,3,4,5,6,7,8,9]. Cardiac involvement typically progresses through three distinct phases: an initial stage of infiltration of eosinophilic cells (necrotic phase); an intermediate thrombotic stage; and a final, late-stage fibrotic phase [1,2,3,4,5,6,7,8,9]. Several myocardial, valvular and vascular abnormalities are known to be associated with HES [10]. Based on the findings, in the left heart, while the left ventricular (LV) volumes typically remain normal, there is a noticeable reduction in the LV global longitudinal strain (LS), apical rotation, and twist, often accompanied by elevated left atrial (LA) volumes and stroke volumes, with its reduced systolic function and mitral annular (MA) dilation and functional impairment. Regarding the right heart, the right ventricular function and volumes are generally preserved, though studies show increased right atrial volumes with mild functional deterioration and a dilated tricuspid annulus (TA) without functional abnormalities. The vascular abnormalities include increased aortic stiffness without dilation and, in certain cases, pulmonary hypertension [10]. Novel cardiovascular imaging techniques like three-dimensional (3D) speckle-tracking echocardiography (STE) are suitable methods for detailed cardiovascular analysis [11,12,13,14]. While the aortic valve is recognized as a pivotal determinant of central hemodynamics, aortic valve annular (AVA) involvement in HES patients has never been examined. Consequently, the objective of this investigation was to evaluate the possible abnormalities in AVA dimensions and dynamics in HES and to assess their associations with function of the LV.

## 2. Methods

**Patient population:** The present retrospective study initially consisted of 17 consecutively enrolled HES patients; however, one participant was removed from the analysis because of poor image quality precluding the accurate measurement of AVA dimensions and LV strains. The final study population comprised 16 HES patients (mean age: 59.7 ± 12.6 years; 11 males). All the HES patients were under the care of the Hematology Center, Department of Medicine, University of Szeged as a tertiary center. They were recruited on a voluntary basis prospectively between 2012 and 2021. Out of 16 HES patients, 13 patients were in the necrotic stage, while 3 were in the fibrotic stage. In the present study, an HES diagnosis was established using one of the most widely accepted criteria [1,2,3,4,5,6,7,8,9]. The findings in the HES patients were compared to those for 21 age- and gender-matched healthy controls (mean age: 54.0 ± 4.9 years; 12 males). They were selected from a pool of more than 300 healthy cases, who were recruited between 2011 and 2017, who voluntarily participated in a complete cardiovascular investigation including a physical examination, laboratory tests, standard 12-lead electrocardiography (ECG), and two-dimensional Doppler echocardiography extended with a 3DSTE data acquisition and analysis at a later date. The clinical evaluations yielded negative results for these subjects, will all the parameters falling within standard reference ranges. The participants were nonsmokers; not taking regular medication; and met the exclusion criteria for obesity (body mass index < 30 kg/m^2^), pregnancy, professional athleticism, or regular yoga practice. Furthermore, no subjects presented with a history of known pathological conditions or disorders. This study is part of the ‘**M**otion **A**nalysis of the heart and **G**reat vessels b**Y** three-dimension**A**l speckle-t**R**acking echocardiography in **Path**ological cases’ (**MAGYAR-Path**) **Study**, which aims to provide detailed analyses of cardiac chambers within specific pathologies. The study protocol was approved by the Institutional and Regional Biomedical Research Committee at the University of Szeged under registration number 71/2011 (latest approval issued on 17 March 2025), and the research adhered to established ethical protocols, with all patients and controls subjects providing their informed consent prior to participation.

**Two-dimensional Doppler echocardiography:** Standard 2D Doppler echocardiography was utilized to evaluate the LA and LV sizes and volumes, alongside the measurement of Simpson’s ejection fraction (EF). Furthermore, the LV diastolic function was assessed via a Doppler analysis, focusing on the calculation of the E/A ratio [15]. Valvular regurgitation and stenosis were ruled out employing color Doppler flow mapping and the calculation of pressure gradients, respectively. Data acquisition was carried out with a Toshiba Artida^TM^ echocardiographic platform utilizing a 1–5 MHz PST-30BT phased-array transducer (Toshiba Medical Systems, Tokyo, Japan).

**Three-dimensional speckle-tracking echocardiography:** Following established clinical protocols, 3DSTE assessments were conducted using the aforementioned ultrasound system, integrated with a 3D-capable PST-25SX matrix-array probe (Toshiba Medical Systems, Tokyo, Japan). The procedure was divided into two parts. First, 3D echocardiographic datasets were obtained via the apical window after optimizing the image quality (gain, magnitude, etc.). Moreover, six subvolumes were acquired over six consecutive cardiac cycles during a single breath-hold, with stable RR intervals on the ECG. The software automatically merged the acquired subvolumes into a full volume. Subsequently, these datasets underwent offline analysis using the vendor-provided 3D Wall Motion Tracking tool (version 2.7, UltraExtend, Toshiba Medical Systems, Tokyo, Japan) [11,12,13,14]. The measurements were averaged over 5 cardiac cycles.

**Figure 1:**
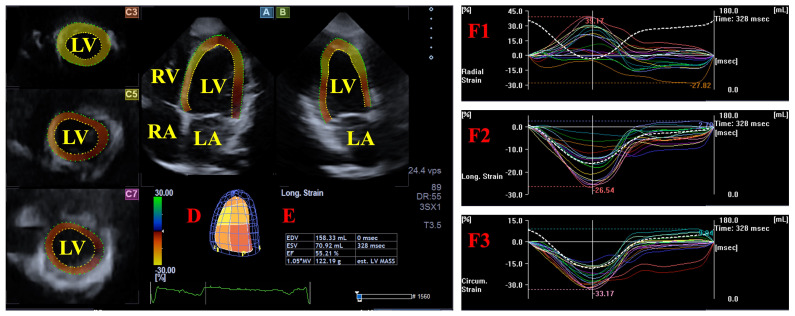
The figure illustrates the imaging planes utilized during the analysis: apical four-chamber view (**A**) and two-chamber (**B**) long-axis views, supplemented by short-axis projections at the apical (**C3**), midventricular (**C5**), and basal (**C7**) levels of the left ventricle (LV). Panel (**D**) depicts a virtual 3D reconstruction of the LV produced by the analysis tool, with the corresponding volumetric data shown in Panel (**E**). Panels (**F1**), (**F2**), and (**F3**) exhibit the global (white line) and segmental (colored lines) time–strain curves for the radial, longitudinal, and circumferential LV strains, respectively, alongside the time–LV volume changes curve (dashed white curve). **Abbreviations.** EDV, end-diastolic volume; EF, ejection fraction; ESV, end-systolic volume; RA, right atrium; RV, right ventricle; LA, left atrium; LV, left ventricle.

For the LV strain analysis, apical four-chamber (AP4CH) and two-chamber (AP2CH) long-axis views were used to automatically generate three cross-sectional views. An observer determined the lateral and septal edges of the LV-MA and the LV apical endocardial contour. After sequential processing and automated border identification, a 3D virtual reconstruction of the LV was generated. Based on this model, the peak unidirectional/unidimensional end-systolic radial (RS), circumferential (CS) and longitudinal (LS) strains were quantified at the basal regional level. These functional parameters represent myocardial thickening/thinning, narrowing/widening, and lengthening/shortening of the LV wall, respectively (Figure 1) [13,16].

**Figure 2:**
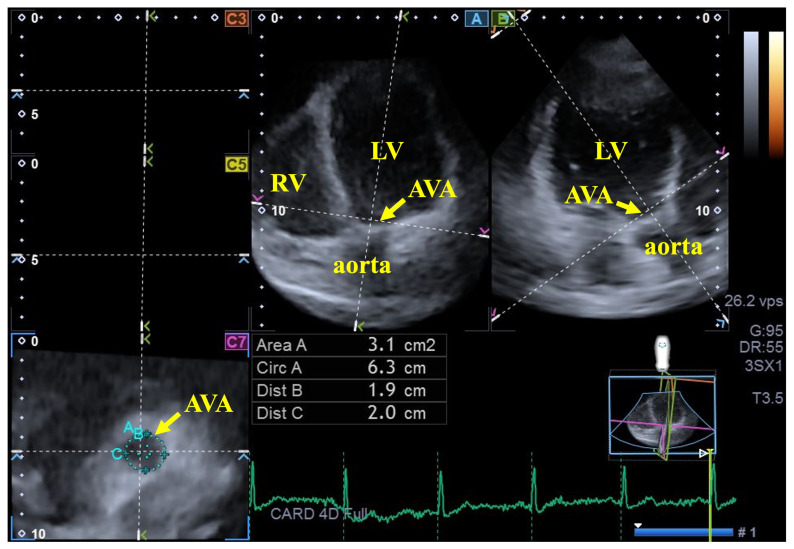
Three-dimensional speckle-tracking echocardiography-derived measurement of the aortic valve annular (AVA) dimensions: apical four-chamber (**A**) and two-chamber (**B**) long-axis views optimized on the AVA and ‘en-face’ view of the AVA in C7 cross-sectional view. **Abbreviations:** LV = left ventricle, AVA = aortic valve annulus, Area = AVA area, Circ = AVA perimeter, Dist B = maximum AVA diameter, Dist C = minimum AVA diameter.

For the AVA dimension measurements, the ideal LV longitudinal planes were established using the AP2CH and AP4CH long-axis views. After the aortic valve and the aorta were visualized and the imaging parameters were optimized by tilting these planes, they were aligned parallel to the centerline of the aortic root. The C7 cross-sectional view—employed for AVA assessment—was oriented perpendicular to the longitudinal plane. Precise care was taken to maintain this perpendicularity, ensuring the exclusion of the Valsalva sinuses and the LV outflow tract from the analysis. The following AVA characteristics were measured at end-diastole (ED) and end-systole (ES)—minimum and maximum AVA diameter (AVA-Dmin and AVA-Dmax), AVA area (AVA-A) and AVA perimeter (AVA-P)—all measured by planimetry [17,18,19]. The AVA plane systolic excursion (AAPSE), defined as the spatial displacement of the AVA plane during the cardiac cycle and considered an analogue of the MAPSE/TAPSE parameters characterizing the spatial displacement of the mitral/tricuspid valve annulus, was also measured (Figure 2) [18,20].

**Statistical Analysis:** The results were expressed as the mean ± standard deviation (SD) or as absolute frequencies and percentages [*n* (%)], depending on the data type. Levene’s test was employed to evaluate the homogeneity of variances. Statistical comparisons were performed using an independent samples *t*-test for the continuous variables and Fisher’s exact test for the categorical data. Pearson correlation coefficients were determined and a multivariable regression analysis was also performed for correlations. The reliability of 3DSTE-based AVA measurements was determined by assessing the intra-observer and inter-observer variability in 21 healthy subjects. These metrics, expressed as the mean ± 2SD of the differences between independent observations, were supplemented by interclass correlation coefficients (ICCs). A post hoc power analysis was performed using G*Power software (version 3.1) based on the observed effect size, an alpha level of 0.05, and the final sample size. Statistical significance was defined at *p* < 0.05, and all computational procedures were executed using SPSS version 29.0.0.0 (SPSS Inc., Chicago, IL, USA).

## 3. Results

**Clinical data:** The clinical characteristics and baseline data for both the HES patients and the healthy control group are presented in Table 1. During their medical evaluation, none of the patients exhibited clinical signs or symptoms typically associated with elevated eosinophil counts. Regarding the medical history of the HES group, no significant cardiovascular events were recorded, with the exception of three specific patients who had myocardial infarction and thromboembolic events in their medical history. Within the HES cohort, several extracardiac manifestations were identified: eosinophilic dermatitis (*n* = 1), duodenal eosinophilia (*n* = 1), and a case of sensorimotor neuropathy accompanied by pulmonary involvement and biopsy-proven granulomatous necrotizing vasculitis (*n* = 1). Additional clinical findings included pulmonary tissue eosinophilia (*n* = 1), isolated skin involvement (*n* = 1), and one instance of acute T-cell lymphoma-associated hypereosinophilia featuring gastrointestinal complications (*n* = 1). Both the HES patient group and control subjects were free of atrial fibrillation, pulmonary embolism, chronic obstructive pulmonary disease, and any coexisting malignancies.

**Two-dimensional Doppler echocardiography**: There were no statistically significant disparities identified between the HES cohort and the healthy control group in the LA and LV parameters measured throughout the cardiac cycle. Similarly, for the parameters reflecting LV function, specifically LV-EF for systolic function and the E/A ratio for diastolic function (Table 2). Regarding valvular function, one HES patient exhibited grade 2 mitral regurgitation; however, all the other subjects, including the remaining HES cohort and the control group, showed no evidence of mitral, tricuspid or aortic regurgitation exceeding grade 1, nor was any significant valvular stenosis detected. 

**Three-dimensional speckle-tracking echocardiographic data:** The AVA measurements were not indexed to the body surface area, as the analysis also sought direct correlations and associations with basal LV strains. Among the 16 HES patients, the end-systolic AVA-A was larger than, equal to, or smaller than the end-diastolic AVA-A in four (25%), three (19%) and nine (56%) cases, respectively. In the matched group, these proportions proved to be 12 (57%), one (5%) and eight (38%), respectively. None of the AVA dimensions measured in end-systole and end-diastole differed significantly between the HES patient group and the matched healthy controls. No significant differences were found in the end-diastolic and end-systolic AVA maximum and minimum AVA diameters, areas or perimeters between the HES cohort and the control group. The AAPSE was significantly reduced in the HES cohort. Among the basal LV strain parameters, only the LV-LS proved to be impaired in the HES patients (Table 3).

**Correlation, multivariable regression and post hoc power analyses:** The AAPSE correlated with the basal LV-RS (r = 0.59, *p* = 0.02) and basal LV-CS (r = −0.61, *p* = 0.03), but showed no correlation with the basal LV-LS (r = −0.25, *p* = 0.20). After a multivariable adjustment for age, sex, body mass index, systolic and diastolic blood pressures, thickness of the interventricular septum, hypertension, diabetes mellitus and hypercholesterolemia, the results remained consistent, confirming the associations were independent of these potential confounders. A post hoc power analysis was performed to evaluate the robustness of the observed difference. With the current sample sizes, the analysis revealed a large effect size (Cohen’s d = 0.8) and an achieved power of 0.65 at an alpha level of 0.05.

**Reproducibility of 3DSTE-derived AVA assessments:** The intra-observer (same examiner measured twice) and inter-observer (two independent examiners) variability for the 3DSTE-derived end-systolic and end-diastolic AVA minimum and maximum perimeters, areas, and diameters and AAPSE are shown in Table 4. The table presents the mean ± 2SD differences in the measured values, along with the respective ICCs.

## 4. Discussion

Cardiac manifestations in HES generally evolve through three distinct sequential phases, beginning with the initial acute necrotic stage, which is often asymptomatic, and is characterized by extensive myocardial eosinophilic infiltration. This is followed by the thrombotic phase, during which thrombus formation occurs, and subsequently organizes into a dense layer of granulation tissue. During the concluding fibrotic stage, this granulation tissue undergoes progressive fibrotic transformation [1,2,3,4,5,6,7,8,9]. The characteristic echocardiographic features of HES involve endocardial fibrosis, fibrothrombotic occlusion of the LV apex, and clinically significant valvular insufficiency [10]. However, the data obtained using advanced imaging techniques in larger series of HES patients remain limited. Regarding the LV, no ventricular dilation, but a reduction in the global LV-LS (predominantly affecting the basal regions), as well as altered apical LV rotation and twist can be detected in the early stages of the disease [10]. According to the literature, no specific AV abnormalities have been demonstrated in HES to date.

A 3DSTE seems to be an appropriate tool for the simultaneous assessment of AVA dimensions and LV strains, using the same acquired 3D echocardiographic datasets and well-defined normal reference values [13,16,17]. This fact makes the methodology appropriate for detailed (patho)physiologic analysis in certain disorders [18].

The clinical implications of our findings can be summarized as follows. First, it is suggested that the dimensions and dynamics of the AVA respecting the heart cycle can be assessed using a relatively simple 3DSTE-based method, by utilizing the identical, digitally captured 3D echocardiographic datasets used for LV strain analysis, even in a rare disease such as HES. Secondly, unlike the atrioventricular valves, the dimensions of the AVA do not show pronounced changes during the cardiac cycle: based on the literature data, a larger AVA area is observed twice as frequently in end-systole as in end-diastole [19]. In the present study, a higher ratio of HES patients exhibited greater end-diastolic AVA compared with the matched healthy controls, suggesting altered AVA dynamics. Thirdly, earlier findings from the MAGYAR-Path Study showed MA dilation in end-diastole and in end-systole in HES, whereas TA dilation was less pronounced [20]. Our current findings extend this observation by showing no significant AVA dilation in a series of patients with HES. Fourthly, only the basal LV-LS proved to be reduced in HES, with preserved LV-RS and LV-CS, suggesting deterioration of LV longitudinal deformation. Moreover, the AAPSE was also reduced, further confirming the fact that AVA dynamics are impaired without AVA dilation in HES. Impaired global LV-LS is known to be an early marker of functional impairment, may contribute to an earlier recognition of cardiac involvement and potentially inform patient management beyond conventional echocardiographic parameters, and is known to be associated with reduced survival in certain disorders [21,22,23,24]. In recent findings from the MAGYAR-Path Study, a reduced 3DSTE-derived global LV-LS could be detected in HES, localized to the basal regions [10]. This knowledge is extended with the presently demonstrated findings. Finally, although not investigated in the present study, the increased aortic stiffness reported in HES may contribute to the partially altered AV dynamics [10]. Theoretically, these results could be explained by eosinophilic infiltration of the AVA itself and/or the surrounding LV/aorta tissue and the resulting change in tissue quality. Moreover, the clinical relevance of findings is not clear and suggests the need for further diagnostic and prognostic studies in a larger HES patient population. It will also be important to clarify whether there is a relationship with the disease stage, or if it could have a role as an early marker preceding overt valvular dysfunction. The presented observations reflect an exploratory pattern, rather than conclusive evidence of structural or functional AVA remodeling. The investigation should be considered hypothesis generating and its conclusions should be evaluated accordingly.

## 5. Limitations Section

The present study is subject to several important limitations that should be considered:

- The image quality of conventional 2D echocardiography remains superior to that of 3DSTE due to technical reasons, such as a higher spatial and temporal resolution. In addition, the greater physical size of a 3DSTE transducer complicates optimal probe positioning. The acquisition procedure, necessitating the integration of six subvolumes over six consecutive cardiac cycles to maximize image resolution, is potentially prone to stitching errors and motion-induced artifacts [11,12,13,14]. If other technical limitations of echocardiography are taken into account, valvular regurgitation is only assessed visually and qualitatively. More advanced, quantitative scoring methods were not applied in the present analysis. Although advanced imaging modalities (such as magnetic resonance imaging, computer tomography or other echocardiographic techniques) may provide additional information on AVA morphology, such detailed assessments were beyond the defined scope of this study.
- This analysis includes data from a limited number of HES patients (*n* = 16). The HES cohort is clinically heterogeneous, including varying extracardiac involvement, cardiovascular risk factors, and prior ischemic or thromboembolic events, all of which may influence the LV strain and AVA dynamics. However, HES is a rare disease, and the data presented in this study originate exclusively from a single center. According to the literature, this study involves the largest number of HES patients studied to date [10]. All these facts should be taken into account when interpreting the findings.
- Due to the low number of involved HES patients, from a statistical point of view the study may be underpowered to detect subtle AVA dimensional differences; therefore, there is the possibility of type II errors, despite adequate reproducibility.

## 6. Conclusions 

In HES patients, the AVA is not significantly dilated. However, a greater end-diastolic AVA area is observed more frequently compared with age- and gender-matched healthy controls. In addition, the aortic annular plane systolic excursion (AAPSE) and basal LV-LS are significantly reduced in the HES cohort.

## Figures and Tables

**Table 1:** **Clinical and laboratory data of patients with idiopathic hypereosinophilic syndrome and** **controls.**

	Controls (*n* = 21)	HES Patients (*n* = 16)	*p*
age (years)	54.0 ± 4.9	59.7 ± 12.6	0.81
male gender (%)	12 (57)	11 (69)	0.47
hypertension (%)	0 (%)	9 (56)	<0.001
diabetes mellitus (%)	0 (%)	1 (6)	0.23
hypercholesterolemia (%)	0 (%)	5 (31)	0.006
white blood cell count (g/L)	6.2 ± 0.9	14.9 ± 6.3	0.03
eosinophil ratio (%)	3.2 ± 2.0	45.2 ± 16.4	0.003
absolute eosinophil count (g/L)	0.2 ± 0.2	7.3 ± 4.3	0.03
red blood cell count (t/L)	4.2 ± 0.4	4.3 ± 0.3	0.79
hemoglobin (g/L)	130.9 ± 9.3	123.0 ± 16.8	0.82
platelet count (g/L)	272.6 ± 162.3	272.9 ± 152.4	0.78
hematocrit (%)	38.3 ± 5.3	36.9 ± 5.1	0.88

**Table 2:** **Two-dimensional echocardiographic data of patients with idiopathic hypereosinophilic syndrome and** **controls.**

	Controls (*n* = 21)	HES Patients (*n* = 16)	*p*
LA diameter (mm)	39.76 ± 4.46	41.81 ± 5.85	0.25
LV end-diastolic diameter (mm)	47.63 ± 3.60	51.32 ± 10.71	0.24
LV end-diastolic volume (mL)	107.01 ± 20.67	114.97 ± 45.13	0.59
LV end-systolic diameter (mm)	31.35 ± 3.23	34.04 ± 11.01	0.35
LV end-systolic volume (mL)	36.08 ± 9.27	42.20 ± 20.47	0.31
Interventricular septum (mm)	9.58 ± 1.37	10.69 ± 1.22	0.01
LV posterior wall (mm)	9.72 ± 1.49	9.74 ± 1.21	0.89
LV ejection fraction (%)	65.88 ± 3.54	63.65 ± 8.72	0.52
E (cm/s)	70.53 ± 16.19	75.14 ± 17.52	0.42
A (cm/s)	72.29 ± 18.21	73.43 ± 15.89	0.88
E/A	1.03 ± 0.29	1.13 ± 0.36	0.49

**Abbreviations:** HES = idiopathic hypereosinophilic syndrome, LA = left atrial, LV = left ventricular, E and A = early and late diastolic mitral inflow velocities.

**Table 3:** **Comparison of three-dimensional speckle-tracking echocardiography-derived aortic valve annular dimensions and aortic valve plane systolic excursion between patients with idiopathic hypereosinophilic syndrome and** **controls.**

	Controls (*n* = 21)	HES Patients (*n* = 16)	*p*
**AVA-Dmax-D (cm)**	2.07 ± 0.26	2.09 ± 0.33	0.78
**AVA-Dmin-D (cm)**	1.88 ± 0.26	1.78 ± 0.39	0.39
**AVA-A-D (cm^2^)**	3.33 ± 0.76	3.13 ± 0.88	0.47
**AVA-P-D (cm)**	6.52 ± 0.76	6.31 ± 0.86	0.44
**AVA-Dmax-S (cm)**	1.98 ± 0.29	1.98 ± 0.26	0.94
**AVA-Dmin-S (cm)**	1.92 ± 0.27	1.75 ± 0.21	0.08
**AVA-A-S (cm^2^)**	3.28 ± 0.84	2.89 ± 0.65	0.15
**AVA-P-S (cm)**	6.41 ± 0.89	6.08 ± 0.68	0.23
**AAPSE (cm)**	1.12 ± 0.24	0.91 ± 0.29	0.05
**basal LV-RS (%)**	34.56 ± 12.20	35.92 ± 15.10	0.89
**basal LV-CS (%)**	−27.32 ± 5.99	−26.30 ± 6.27	0.86
**basal LV-LS (%)**	−21.62 ± 4.76	−16.63 ± 4.99	0.03

**Abbreviations:** HES = idiopathic hypereosinophilic syndrome, AVA = aortic valve annulus, Dmax = maximum diameter, Dmin = minimum diameter, A = area, P = perimeter, D = end-diastolic, S = end-systolic, AAPSE = aortic valve plane systolic excursion. Basal LV strains and AVA values are derived from same 3D echocardiographic datasets.

**Table 4:** **Intra- and inter-observer variability in three-dimensional speckle-tracking echocardiography-derived assessment of aortic valve annular dimensions and aortic valve plane systolic** **excursion.**

	Intra-Observer Agreement		Inter-Observer Agreement	
	Mean ± 2SD Difference in Values Obtained for 2 Measurements of the Same Observer	Correlation Coefficient Between Measurements of the Same Observer	Mean ± 2SD Difference in Values Obtained by 2 Observers	Correlation Coefficient Between Independent Measurements of 2 Observers
**AAPSE (cm)**	−0.03 ± 0.18	0.91 (*p* < 0.01)	−0.03 ± 0.23	0.91 (*p* < 0.01)
**AVA-Dmax-D (cm)**	−0.04 ± 0.16	0.89 (*p* < 0.01)	−0.05 ± 0.15	0.89 (*p* < 0.01)
**AVA-Dmin-D (cm)**	−0.02 ± 0.22	0.89 (*p* < 0.01)	−0.04 ± 0.26	0.92 (*p* < 0.01)
**AVA-A-D (cm^2^)**	−0.12 ± 0.63	0.93 (*p* < 0.01)	−0.10 ± 0.52	0.93 (*p* < 0.01)
**AVA-P-D (cm)**	−0.05 ± 0.63	0.92 (*p* < 0.01)	−0.11 ± 0.67	0.93 (*p* < 0.01)
**AVA-Dmax-S (cm)**	0.02 ± 0.35	0.92 (*p* < 0.01)	0.04 ± 0.37	0.94 (*p* < 0.01)
**AVA-Dmin-S (cm)**	0.05 ± 0.32	0.83 (*p* < 0.01)	0.03 ± 0.41	0.85 (*p* < 0.01)
**AVA-A-S (cm^2^)**	0.13 ± 0.67	0.90 (*p* < 0.01)	0.11 ± 0.69	0.94 (*p* < 0.01)
**AVA-P-S (cm)**	−0.02 ± 0.60	0.93 (*p* < 0.01)	0.01 ± 0.62	0.92 (*p* < 0.01)

**Abbreviations:** AVA = aortic valve annulus, Dmax = maximum diameter, Dmin = minimum diameter, A = area, P = perimeter, D = end-diastolic, S = end-systolic, AAPSE = aortic valve plane systolic excursion.

## Data Availability

The original contributions presented in this study are included in the article. Further inquiries can be directed to the corresponding author.
